# Malaria microscopy evaluation and quality assurance in rural clinics of Rarieda and Alego Usonga sub-counties of Siaya County, western Kenya

**DOI:** 10.1186/s12936-026-05886-0

**Published:** 2026-04-09

**Authors:** Jonathan S. Schultz, Oliver Towett, Kizito Obiet, Brian Seda, Kephas Otieno, Dennis Omondi, Wycliffe Odongo, Victoria Seffren, Philemon Wafula, Eunice Oreri, Daniel P. McDermott, Sarah G. Staedke, Titus K. Kwambai, Simon Kariuki, Julie R. Gutman

**Affiliations:** 1 Malaria Branch, Division of Parasitic Diseases and Malaria, US Centers for Disease Control and Prevention, Kisumu, Kenya; 2 Centre for Global Health Research, Kenya Medical Research Institute, Kisumu, Kenya; 3 Malaria Branch, Division of Parasitic Diseases and Malaria, US Centers for Disease Control and Prevention, Atlanta, GA USA; 4 County Department of Health, Siaya, Kenya; 5 Department of Vector Biology, Liverpool School of Tropical Medicine, Liverpool, UK

**Keywords:** Plasmodium falciparum, Malaria, Microscopy, Diagnostics, Parasitemia, Quality assurance

## Abstract

**Background:**

Although rapid diagnostic tests (RDTs) are widely used for malaria diagnosis, they have notable limitations. Blood smear (BS) microscopy remains the gold standard, yet its reliability in public health facilities (HFs) across malaria-endemic regions of Kenya can be compromised by limited infrastructure, technical capacity, and quality assurance.

**Methods:**

We assessed the quality of malaria microscopy in 29 HFs in Siaya County, western Kenya, from January–July 2024 by evaluating the concordance of routine HF BS results with expert microscopy. We evaluated the availability and quality of reagents, standard operating procedures, and infrastructure using the National Malaria Control Program (NMCP) technical supervision checklist which follows WHO-certified microscopy standards. Up to 60 participant slides were randomly selected over three visits and two slides were prepared for each participant. Slide 1 was prepared and read on-site as per routine HF practice, then re-examined by an expert microscopist using the HF microscope and again at the Kenya Medical Research Institute (KEMRI). Slide 2 was stained and read at KEMRI, serving as the gold standard. We evaluated factors and characteristics associated with accurate diagnosis using logistic regression.

**Results:**

Of the 1,494 blood smears examined, 501 (34%) were positive. Concordance between routine microscopy and expert re-reading was 91% (1,289/1,414), ranging from 55% (6/11) to 100% (60/60) across health facilities. Percent agreement between HF slide 1 and slide 2 was 86% (1,276/1,485), with a range from 55% (6/11) to 98% (39/40) by HF. Compared to slide 2, sensitivity and specificity of HF results was 76% and 92%, respectively, resulting in undertreatment of 24% and overtreatment of 8% of patients. Those with parasitemia 1–100 p/μL had lower odds of accurate diagnosis (OR = 0.12; 95% CI: 0.05–0.29; p < 0.001) while specimens with parasite densities > 10,000 p/μL had higher odds of accurate diagnosis (OR = 6.08; 95% CI: 2.22–25.1; p = 0.002), indicating a positive association between parasite density and diagnostic accuracy. Six (21%) of HFs had poor quality BS with debris or contamination.

**Conclusions:**

While overall concordance was high, the variability in results by HF, limited accuracy at low parasite densities, and challenges with required infrastructure highlight the need for ongoing malaria microscopy quality assurance to ensure proper case management.

**Supplementary Information:**

The online version contains supplementary material available at 10.1186/s12936-026-05886-0.

## Background

Globally, there were 282 million cases of malaria and 610,000 malaria deaths in 2024, an increase from 263 million cases of malaria and 597,000 malaria deaths in 2023 [1, 2]. Approximately 95% occurred in the WHO African Region, where many at risk still have limited access to timely services for prevention, detection, and treatment of malaria [2]. In Kenya, approximately 70% of the population is at risk for the disease [3, 4]. Malaria accounted for approximately 6 million healthcare visits in Kenya in 2023, representing almost 21% of all outpatient consultations [3, 4].

Parasitological diagnosis of malaria with quality assured rapid diagnostic tests (RDTs) or light microscopy of blood smears (BS) is recommended by the World Health Organization (WHO). Light microscopy remains the gold standard for malaria diagnosis across sub-Saharan Africa [5]. Microscopy enables species identification (*Plasmodium falciparum*, *P. vivax*, *P. malariae*, *P. ovale* and *P. knowlesi*) and quantification of parasite density, which are critical for assessing disease severity, appropriate treatment, and monitoring therapeutic response [6]. HRP2-based RDTs are limited as patients can remain positive for several weeks after parasite clearance due to persistent HRP2 antigenemia, resulting in false positive RDT results [7]. However, BS microscopy remains challenging in public health facilities (HF) due to suboptimal reagents, limited equipment and technical capacity, inadequate infrastructure (electricity, equipment maintenance, and clean water). High quality microscopy can also be time intensive. These challenges threaten the ability to reliably provide timely and accurate results in HFs in malaria endemic areas. To support diagnostic accuracy and consistency, the WHO provides standardized protocols, bench aids, and external quality assurance (EQA) recommendations aimed at strengthening routine malaria microscopy services [6]. A simple EQA pilot program conducted in Kenya from January to February 2014 resulted in improved concordance of BS reads at HFs with expert reads from 77% in non-EQA pilot facilities to 93% of slide results from EQA-pilot facilities (p < 0.001) [8]. They also showed that more recently trained microscopists in EQA-pilot facilities performed better on microscopy performance measures with 97% sensitivity and 100% specificity compared to those in non-EQA pilot facilities (69% sensitivity; 93% specificity; p < 0.01).

Both microscopy and RDTs are recommended for malaria diagnosis before treatment in Kenya [9]. While RDTs are commonly used at lower-tier health facilities and by community health workers (CHWs), Kenya is unique in that nearly half of all malaria diagnoses are still made using blood smear microscopy (BS), despite multiple limitations to performing BS in public HFs [4, 10, 11]. Additionally, in Kenya approximately 40–50% of patients seek initial care for malaria in the private sector, typically in unregulated drug outlets/pharmacies, where adherence to the national malaria diagnostic and treatment guidelines is not guaranteed and patients may receive inaccurate diagnosis and substandard care [12]. Participation in structured external quality assurance (EQA) programs organized by national laboratory programs is recommended by WHO and has been shown to enhance the accuracy of microscopy; however, the EQA program in western Kenya is not optimally implemented [8]. This gap is largely attributable to inconsistent funding and varying levels of support for EQA, as well as capacity to perform Technical Training and Supervision (TSS) site visits across the country [4, 8].

The Kenya Medical Research Institute (KEMRI), and the U.S Centers for Disease Control and Prevention (CDC) have collaborated in conducting epidemiological and entomological malaria research in western Kenya since 1979, with the addition of the Liverpool School of Tropical Medicine (LSTM) to the research collaboration in 2013. The partnership works alongside the Siaya County Department of Health and the National Malaria Control Program (NMCP) to support malaria surveillance activities in Rarieda and Alego Usonga sub-counties, Siaya County, including supporting routine surveillance data collection at selected HFs and using HF incidence estimates as an outcome for the evaluation of malaria control interventions and recent vector control trials [13, 14]. To ensure that the results documented in the routine registers are accurate, ongoing EQA of diagnostic testing including microscopy quality evaluation, as recommended by the WHO and MOH, is critical. Thus, an assessment of microscopy quality was undertaken on the diagnostic performance of routine HF microscopy, the variability between HFs, and the factors associated with accurate diagnosis, as well as identifying local challenges with performing malaria microscopy in Siaya County, western Kenya.

## Methods

### Study setting and malaria epidemiology

From January to July 2024, we evaluated the diagnostic performance of BS results with WHO-certified expert microscopy at 29 randomly selected HFs in Alego Usonga and Rarieda sub-counties, which are two of six sub-counties of Siaya County, western Kenya. This is an area of moderate to high malaria endemicity that has been the site of many malaria surveillance programmes [15], evaluation of vector interventions and clinical trials and has been previously described in detail [16–22]. *P. falciparum* is the dominant malaria species with consistently high perennial transmission, with typically peak transmission occurring in May–July and October–November, following the long and short rains respectively [16].

### Health facility distribution

There are currently 8 level 4 hospital facilities in Alego Usonga and Rarieda sub-counties including the Siaya County Referral Hospital, 32 level 3 facilities, typically sub-county hospitals, and 65 level 2 facilities which are lower level public health facilities that include health centers and dispensaries [23].

### Participant selection and sample size calculation

At each public HF, we randomly selected up to 60 patients of any age attending the outpatient departments (OPD) and antenatal care (ANC) of the HFs with suspected malaria for evaluation over 3 different supervision visits. The expected number of 60 participants per facility was determined to detect a difference between two readers with an assumed sensitivity of 70% and 90%, with a type I error rate of 0.05 (two-tailed) and type II error rate of 0.20 [24].

### Laboratory procedures and quality assurance

Two BS were collected by HF staff as part of routine clinical care; the first was prepared and read on-site by HF staff following their routine procedures (Routine S1), then re-read by an on-site KEMRI Expert microscopist (certified by WHO) during an EQA visit using the HF microscope (Expert S1). The same slide was re-examined again by an expert microscopist at the KEMRI Malaria Laboratory (KEMRI S1). A second BS (S2) was collected, prepared by the HF staff and dried at the HF, but not stained; this slide was stained and read at the KEMRI Malaria Laboratory, using quality assured reagents, and used as the gold standard for comparisons [6]. Expert re-reads of S1, S2, and health facility results were all blinded between microscopists. At each HF, the quality of reagents, slide preparation, availability of standard operating procedures (SOPs), and infrastructure (electricity and microscope) were documented using the Kenyan NMCP published TSS checklist (Supplement).

#### Analysis

Data were directly entered into an ODK database during HF visits and during microscopy reading by the microscopists at the KEMRI Malaria Laboratory and analyzed using R Project for Statistical Computing (RRID:SCR_001905), R version 4.0.2. The sensitivity, specificity, positive predictive value (PPV), negative predictive value (NPV), and percent agreement were determined between the routine HF results and expert re-reads at the HF and at KEMRI. Percent agreement was determined between Routine S1 and S2 by level of parasitemia from the gold standard (S2) to evaluate how performance measures changed by parasite density. The relationship between participant characteristics, including sex, age (under 5 years vs. 5 and over), parasite density (categorized into 5 groupings: negative (0 p/μl, reference group), 1–99 p/μl, 100–999 p/μl, 1,000–9,999 p/μl, ≥ 10,000 p/μl) was assessed. The parasite density categories correspond approximately to 0%, 0.002–0.025%, 0.025–0.25%, 0.25–2.5%, > 2.5% parasitemia, respectively [25]. Facility characteristics, included sub-county location, laboratory staff qualifications (degree versus diploma), and facility level 2, 3, or 4 (categorized as an ordinal variable with Level 2 as reference), and accuracy of malaria diagnosis were evaluated using univariable and multivariable logistic regression.

#### Ethics

Informed written consent was obtained from all participants (or parent/guardian of participants aged < 18), and informed written assent was obtained from all participants aged 13 to 17. For illiterate participants, consent or assent was obtained through impartial witness attestation, whereby the consent form was read aloud in the participant’s preferred language (English, Kiswahili, or DhuLuo), and the witness confirmed that the information was accurately conveyed and that consent was freely given; the illiterate participant then provided a thumbprint in lieu of a signature. Consent and assent were obtained in either English, Kiswahili, or DhuLuo according to the participant’s preference. The health facility surveillance protocol was reviewed and approved by KEMRI (#2809), CDC (#6733) and LSTM (#15-003) Institutional Review Boards.

## Results

From January to July 2024, a total of 1,494 participants seeking care at OPD and ANC were recruited at 29 HFs and had blood smears collected and evaluated (Table 1). Most patients were over 5 years of age (75%), female (64%), and seen in outpatient departments (76%); 13% were seen at a hospital (Level 4), 50% at a Health Center (Level 3), and 37% at a dispensary (Level 2). Among all slides read, 19% were read by microscopists who had a degree and 81% by those who had a diploma as their highest level of training in laboratory science (Table 1). Among the 1494 blood smears, 80 (5%) were unable to be re-read at the time of the EQA visit due to the poor quality of either the smear or the available microscope, and 36 (2%) were unavailable to be re-read at the KEMRI malaria laboratory due to the poor quality of the smear.

**Table 1:** Participant and health facility (HF) characteristics that participated in microscopy quality assurance evaluation in Rarieda and Alego Usonga sub-counties in western Kenya

Participant characteristics	Alego Usonga, N = 910	Rarieda, N = 584	Overall, N = 1,494
Sex			
Female	594 (65.3%)	359 (61.5%)	953 (63.8%)
Age			
< 5 years	210 (23.1%)	103 (17.6%)	313 (21.0%)
5–17 years	323 (35.5%)	248 (42.5%)	571 (38.2%)
** ≥ **18 years	377 (41.4%)	232 (39.7%)	609 (40.8%)
Health Facility Characteristics			
Health Facility (HF) Department			
Antenatal Care (ANC)	20 (2.2%)	27 (4.6%)	47 (3.1%)
Outpatient Department (OPD) Over 5	682 (74.9%)	456 (78.1%)	1,138 (76.2%)
Outpatient Department (OPD) Under 5	208 (22.9%)	101 (17.3%)	309 (20.7%)
Level of Health Facility			
Dispensary (Level 2)	331 (36.4%)	73 (12.5%)	505 (33.8%)
Health Centre (Level 3)	399 (43.8%)	95 (16.3%)	689 (46.1%)
Hospital (Level 4)	120 (13.2%)	12 (2.1%)	180 (12.0%)
Microscopists Training Level			
Degree	189 (20.8%)	99 (17.0%)	288 (19.3%)
Diploma	721 (79.2%)	485 (83.0%)	1,206 (80.7%)

The overall routine HF microscopy test positivity rate was 34% (501/1,494). When re-read by expert microscopists using the facility-prepared slides and microscope at the HF (Expert S1) positivity was 32% (457/1,414); when re-read at the KEMRI laboratory (KEMRI S1) positivity was 31% (454/1,458), using the KEMRI lab-stained slide (S2), positivity was 37% (552/1,485) (Tables 2 and 3). Compared to S2 as the reference gold standard, the routine HF results showed a sensitivity of 76%, specificity of 92%, positive predictive value (PPV) of 85%, and negative predictive value (NPV) of 87%, and overall percent agreement of 86% (Table 3). Among participants with a negative gold standard BS (S2), a total of 77/933 (8%) were incorrectly diagnosed with malaria (false positives), and among participants with a positive gold standard BS (S2), a total of 132/552 (24%) were not diagnosed with malaria (false negatives) at the HFs. This gives an overall percent agreement of 86% between the HF read and the gold standard.

**Table 2:** Malaria microscopy positivity by participant and health facility (HF) characteristics using routine health facility results and expert re-read in Rarieda and Alego Usonga sub-counties in western Kenya. Results reported are microscopy positive/total participants (% positive) by characteristic

Participant characteristics	Alego Usonga, N = 910	Rarieda, N = 584	Overall, N = 1494
Sex			
Female	174/594 (29.3%)	104/359 (29%)	278/953 (29.2%)
Male	122/316 (38.6%)	101/225 (44.9%)	223/541 (41.2%)
Age			
< 5 years	81/210 (38.6%)	27/103 (26.2%)	108/313 (34.5%)
5–17 years	150/323 (46.4%)	126/248 (50.8%)	276/571 (48.3%)
>= 18 years	65/377 (17.2%)	52/232 (22.4%)	117/609 (19.2%)
Total Positive (Routine S1)	296/910 (33%)	205/584 (35%)	501/1494 (34%)
Health Facility Characteristics			
Health Facility (HF) Department			
Antenatal Care (ANC)	4/20 (20%)	1/27 (3.7%)	5/47 (10.6%)
Outpatient Department (OPD) Over 5	212/682 (31.1%)	177/456 (38.8%)	389/1138 (34.2%)
Outpatient Department (OPD) Under 5	80/208 (38.5%)	27/101 (26.7%)	107/309 (34.6%)
Level of Health Facility			
Dispensary (Level 2)	140/331 (42.3%)	73/174 (42%)	213/505 (42.2%)
Health Centre (Level 3)	125/399 (31.3%)	95/290 (32.8%)	220/689 (31.9%)
Hospital (Level 4)	15/120 (12.5%)	12/60 (20%)	27/180 (15%)
Microscopists Training Level			
Degree	56/189 (29.6%)	31/99 (31.3%)	87/288 (30.2%)
Diploma	240/721 (33.3%)	174/485 (35.9%)	414/1206 (34.3%)
Microscopy results			
Parasite Density: Health Facility (S1)	N = 859	N = 523	N = 1382
Negative	566 (66%)	318 (61%)	884 (64%)
Positive	293 (33%)	205 (35%)	498 (34%)
1–100 p/μl	15 (1.7%)	9 (1.7%)	24 (1.7%)
100–1000 p/μl	115 (13%)	65 (12%)	180 (13%)
1000–10,000 p/μl	92 (11%)	83 (16%)	175 (13%)
>10,000 p/μl	71 (8.3%)	48 (9.2%)	119 (8.6%)
Unavailable^a^	51	61	112
Parasite Density: Expert at HF (S1)	N = 876	N = 518	N = 1374
Negative	599 (68%)	358 (72%)	957 (70%)
Positive	297 (33%)	160 (31%)	457 (32%)
1–100 p/μl	12 (1.4%)	5 (1.0%)	17 (1.2%)
100–1000 p/μl	75 (8.6%)	31 (6.2%)	106 (7.7%)
1000–10,000 p/μl	77 (8.8%)	35 (7.0%)	112 (8.2%)
>10,000 p/μl	113 (13%)	69 (14%)	182 (13%)
Unavailable^a^	34	86	120
Parasite Density: KEMRI Slide 1	N = 834	N = 526	N = 1360
Negative	615 (74%)	389 (74%)	1004 (74%)
Positive	219 (32%)	137 (30%)	356 (31%)
1–100 p/μl	10 (1.2%)	0 (0%)	10 (0.7%)
100–1000 p/μl	49 (5.9%)	28 (5.3%)	77 (5.7%)
1000–10,000 p/μl	64 (7.7%)	26 (4.9%)	90 (6.6%)
>10,000 p/μl	96 (12%)	83 (16%)	179 (13%)
Unavailable^a^	76	58	134
Parasite Density: KEMRI Slide 2	N = 889	N = 576	N = 1465
Negative	577 (65%)	356 (62%)	933 (64%)
Positive	312 (36%)	220 (39%)	532 (37%)
1–100 p/μl	17 (1.9%)	4 (0.7%)	21 (1.4%)
100–1000 p/μl	60 (6.7%)	25 (4.3%)	85 (5.8%)
1000–10,000 p/μl	81 (9.1%)	58 (10%)	139 (9.5%)
>10,000 p/μl	154 (17%)	133 (23%)	287 (20%)
Unavailable^a^	21	8	29

^a^Unavailable microscopy participant results were due to missing slides or poor-quality deemed unreadable by the microscopists

^b^n (%)

**Table 3:** Malaria blood smear microscopy results comparing the routine health facility (Routine S1) to expert re-read of the same saved clinical slide (Expert S1) at the facility using the facility microscope, compared to expert re-read at the KEMRI Malaria Laboratory with high quality microscope (KEMRI S1), compared to a second saved clinical slide stained and read at KEMRI Malaria Laboratory (S2), which was considered the gold standard

Comparison	Test result	KEMRI S2 Positive	KEMRI S2 Negative	Unavailable^a^	Total	Sensitivity	Specificity	Percent agreement
Routine S1	Positive	420	77	4	501	76%	92%	86%
	Negative	132	856	5	993			
Expert S1	Positive	411	42	4	457	79%	95%	85%
	Negative	106	846	5	957			
	Unavailable^a^	35	45	0	80			
KEMRI S1	Positive	409	41	4	454	76%	95%	86%
	Negative	131	868	5	1004			
	Unavailable^a^	12	24	0	36			
Total		552	933	9	1494			

^a^Unavailable microscopy results were due to missing slides or poor-quality deemed unreadable by the microscopists

Higher parasite densities were associated with higher percent agreement: 91% agreement for > 10,000 p/μl, compared to 14% agreement for 1–100 p/μl (Table 4). Facility-level concordance varied widely, with some facilities showing concordance as low as 55% (Supplement Table 1). Despite having a result recorded in the MOH register, 80/1,494 (5%) slides could not be re-read at the HF due to poor slide quality (artifacts, debris, or non-adherence of blood spot due to poor adherence to the slide) or the slide was missing.

**Table 4:** Routine health facility microscopy (Routine S1) testing characteristics and percent agreement stratified by parasitemia from expert read the KEMRI Malaria Laboratory (S2)

	Health Facility results (Routine S1) vs KEMRI Expert S2					
Parasite density (KEMRI Expert S2)	True positive	True negative	False positive	False negative	Percent agreement	Total
Negative	0 (0%)	856 (92%)	77 (8%)	0 (0%)	92%	933
1–99 p/ul	3 (14%)	0 (0%)	0 (0%)	18 (86%)	14%	21
100–999 p/ul	38 (45%)	0 (0%)	0 (0%)	47 (55%)	45%	85
1000–9999 p/ul	106 (76%)	0 (0%)	0 (0%)	33 (24%)	76%	139
≥ 10,000 p/ul	261 (91%)	0 (0%)	0 (0%)	26 (9%)	91%	287
Total	N = 408	N = 856	N = 77	N = 124	86%	1,465

^a^Unavailable microscopy results from 29 participants were due to missing slides or poor-quality deemed unreadable by the microscopists

As expected, when both reads were conducted by expert microscopists (Expert S1 vs KEMRI S1), we saw the highest concordance, at 93% (792/856), excluding slides that could not be read at KEMRI due to poor quality or non-adherence, or missing slides.

### Technical training and supervision results

Infrastructure and quality control program interruptions were noted. Only 3 of 29 facilities had microscopists trained in the last 3 years, and no HF were currently enrolled in an external quality assurance (EQA) program at the time of this activity. Facility stock-outs of supplies and reagents lasting at least 7 days in the prior 3 months were common; 18/29 (62%) had been stocked out of Giemsa stain, 11/29 (38%) had a stock out of microscopy slides or methanol, 15/29 (52%) had a stock out of filter papers to filter the Giemsa stain which was prepared on site at each HF, 16/29 (55%) reported frequent power outages lasting greater than 3 h, and 1/29 (3%) did not have a functioning microscope. Based on expert microscopists evaluation of the quality of blood smears, 6/29 (21%) of facilities had poor quality smears such that it interfered with providing accurate results. Expert readers noted that parasites were often masked by artifacts or debris in 508/1494 (34%) slides read, likely from using unfiltered stain or contaminated water to rinse the slide during preparation. A small portion 9/1494 (0.6%) of thick smears that had been sloughed off (non-adhered) and were thus unable to be re-read by a KEMRI expert (Table 3).

### Factors associated with accurate diagnosis

In univariable and multivariable logistic regression (Table 5), parasite density was the strongest predictor of accurate diagnosis between the routine HF results and gold standard (S2). Using negative slides as the reference group, the odds of accurate diagnosis defined as concordant BS results between the HF and S2, were significantly lower for low-density infections (1–99 p/μl: aOR = 0.18; 95% CI: 0.07–0.45; p < 0.001) and moderate density infections (100–999 p/μL: aOR = 0.63; 95% CI: 0.40–1.00; p = 0.04) and increased as parasitemia increased(1000–9999 p/μL aOR = 1.58; 95%CI 0.90–2.9; p = 0.13; ≥ 10,000 p/μl: aOR = 6.08; 95% CI: 2.22–25.1; p = 0.002) (Table 5).

**Table 5:** Participant and health facility (HF) descriptive characteristics associated with accurate malaria diagnosis, defined as concordant results between the HF and KEMRI S2. Univariable odds ratios (OR) and 95%Cis from logistic regression

Characteristic	Univariable			Multivariable		
	OR^a^	95% CI^a^	p-value	aOR^a^	95% CI^a^	p-value
Sex						
Female	–	–		–	–	
Male	0.99	0.73–1.35	> 0.9	1.03	0.73–1.48	0.9
Age (years)	1.00	0.99–1.01	0.6	1.00	0.99–1.01	> 0.9
Subcounty						
Alego Usonga	–	–		–	–	
Rarieda	1.05	0.78–1.42	0.8	0.95	0.68–1.35	0.8
Staff qualification						
Degree	–	–		–	–	
Diploma	1.05	0.72–1.50	0.8	1.09	0.69–1.69	0.7
Parasitemia density						
Negative (ref)	–	–		–	–	
1–99 p/ul	0.12	0.05–0.29	< 0.001	0.18	0.07–0.45	< 0.001
100–999 p/ul	0.46	0.31–0.69	< 0.001	0.63	0.40–1.00	0.04
1000–9999 p/ul	1.45	0.86–2.60	0.2	1.58	0.90–2.94	0.13
> 10,000 ul	5.65	2.08–23.2	0.004	6.08	2.22–25.1	0.002
Facility level						
Level 2 (ref)	–	–	–	–	–	–
Level 3	1.34	0.97–1.86	0.08	1.12	0.78–1.61	0.5
Level 4	1.49	0.91–2.55	0.13	1.16	0.67–2.10	0.6

^a^OR = Odds Ratio, CI = Confidence Interval

While not statistically significant in either univariable or multivariable models, higher level HFs were slightly better at diagnosing malaria than lower-level facilities. These effects were larger in univariable (Level 3: OR = 1.34; 95% CI: 0.97–1.86, p = 0.08; Level 4: OR = 1.49; 95% CI: 0.91–2.55, p = 0.13) than multivariable analysis (Level 3 aOR = 1.12; 95% CI: 0.78–1.61; p = 0.5; Level 4 aOR = 1.16; 95% CI: 0.67–2.10; p = 0.6). No significant differences in diagnostic accuracy were found by patient sex, age, subcounty, or microscopy staff qualifications.

## Discussion

This simple EQA assessment found 86% overall agreement between results obtained through routine HF BS and expert microscopy. However, these discrepancies resulted in failure to diagnose 24% of patients with malaria (under diagnosis), while another 8% of patients were incorrectly diagnosed with malaria (over diagnosis). These results suggest that if treatment was prescribed and taken by patients appropriately resulting concerning under and over treatment. These results are similar to those previously described in Kenya [8]. Most patients who received incorrect diagnoses had low density parasitemia, demonstrating that additional training and QA support should focus on detection of low-density infections. Ideally, there would be no under or over treatment, and the concordance between routine and expert microscopy for tests dictating clinical care should be > 90% [26]. In addition, 21% of HFs had poor quality slides which precluded rereading, highlighting the need to ensure that slide preparation and staining adhere to standard protocols to facilitate accurate readings. Despite the many challenges, and the need for ongoing EQA, HFs were able to conduct BS microscopy with moderately acceptable diagnostic accuracy.

As expected, this analysis highlights that parasite density is an important predictor of accurate malaria microscopy diagnoses. Slides with low parasite densities (< 1,000 p/μL) were significantly more likely to be misclassified, while those with very high densities (> 10,000 p/μL) were much more likely to be diagnosed correctly. These findings are consistent with established limitations of light microscopy, which has reduced sensitivity at low parasitemia levels and greater reliability at higher parasite loads [5]. This emphasizes the importance of EQA protocols that focus on improving detection thresholds in routine settings [26].

Contrary to expectations, health facility level, whether dispensary (Level 2), health center (Level 3), or sub-county hospital (Level 4), was not significantly associated with diagnostic accuracy. The fact that higher-level facilities did not consistently outperform lower-level ones underscores the potential for quality improvement across all tiers of healthcare levels. The lack of significant effect is likely due, at least in part, to the relatively small sample size in each group, but may also reflect variability in workload and supervision, which can influence performance regardless of facility classification.

Diagnostic accuracy was not associated with staff training/qualification, facility, subcounty, or patient demographics (age or sex). Thus, systematic and technical aspects, rather than patient or staff-level characteristics, are most likely critical to improving diagnostic performance.

Together, these results support targeted strategies to improve malaria BS microscopy, particularly by strengthening diagnostic capacity for low-density infections. This can be done by implementing EQA programs and closely following the WHO standard operating procedures, such as using distilled water with the proper pH when preparing the Giemsa stain and filtering the stain appropriately to prevent debris from contaminating the slides. It is also important to ensure that high quality slides are procured and that only clean slides are used to promote adherence of blood on the slide. Training and supervision programs, such as TSS and EQA programs may benefit from emphasizing detection of low-density infections as these are the infections likely to be missed. In addition, greater reliance on alternative diagnostic tools, such as RDTs, may be required if there are not resources to support EQA or in low performing HFs.

Despite the availability of WHO and national guidelines on QC and EQA of malaria diagnosis, few countries implement them effectively due to various factors including funding limitations, logistical issues, and lack of expertise [26].

This study was restricted to only 29 HFs, all of which were in two sub-counties of Siaya county, where malaria transmission is high and holoendemic. Only three supervision visits were completed at each facility from January to July 2024, when slides were randomly selected and thus results could be different from other times of the year. This limits our ability to generalize the results to areas of Kenya where malaria is less common [3]. Lastly, the lack of appropriate equipment or sub-optimal supplies at some HFs limit the comparisons between microscopists, but highlight the more systematic shortcomings for appropriate BS microscopy.

## Conclusions

Using the NMCP and Supportive Supervision annual checklist, the challenges of performing microscopy at 29 facilities in Siaya County were documented. Facility stock-outs of reagents lasting at least 7 days in the prior 3 months were common, as were power outages which impacted the ability to perform microscopy. Among the 29 participating facilities, all had at least one issue which interfered with their ability to continuously provide accurate microscopy results. In addition, comparison with expert microscopy reads highlighted that while overall concordance between results was high, there were gaps in several facilities which resulted in inappropriate diagnosis and therefore, treatment of patients. Ongoing malaria microscopy quality assurance at public HFs in the study area is essential to ensure that malaria case management is optimized, specifically in areas with highly endemic transmission where HRP2-based RDTs may result in frequent false positives and over estimation of disease burden.

## Supplementary Information

Additional file1

## Data Availability

Data are available upon request from the corresponding author and approval from the CDC, Kenya Medical Research Institute (KEMRI), and Siaya County Department of Health.
