# Supplementary material for: Malaria microscopy evaluation and quality assurance in rural clinics of Rarieda and Alego Usonga sub-counties of Siaya County, western Kenya
Source: Malar J. 2026 Apr 9;25:211. doi: 10.1186/s12936-026-05886-0 (PMC13181933; doi:10.1186/s12936-026-05886-0)
Supplement: Supplementary file 1 — Additional file1 [file 12936_2026_5886_MOESM1_ESM.docx]

**SUPPLEMENTAL MATERIAL**

**Supplemental Figure 1:** Scatter plots with correlations between health facility reported parasitemia concentrations and expert re-read of slide 1 at the facility, expert re-read at the KEMRI Malaria Laboratory, and expert read of slide 2 prepared at the KEMRI Malaria Laboratory.


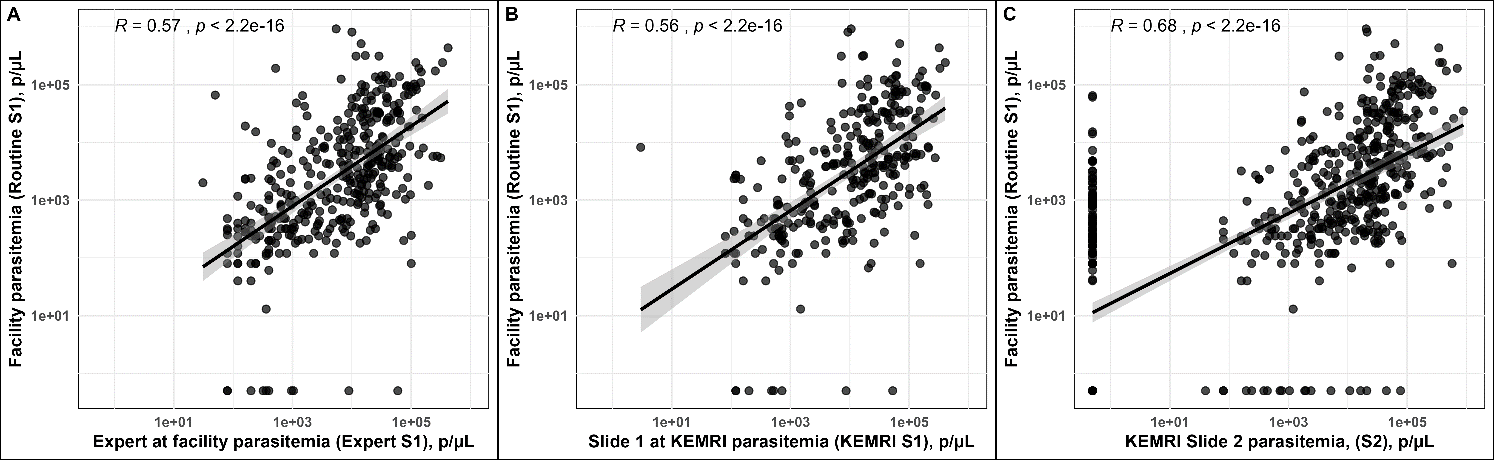


**Supplemental Table 1:** Diagnostic testing accuracy of positivity of blood smear microscopy of comparing the health facility register results to a second prepared and dried blood smear on high quality slide (slide 2) and stained and read at the KEMRI Malaria Laboratory as the gold standard.

|  | **Health Facility compared to Gold Standard Microscopy** | | | | | **Total** |
| --- | --- | --- | --- | --- | --- | --- |
|  | True Positive | True Negative | False Positive | False Negative | Unknown |  |
| **Health facility** |  |  |  |  |  |  |
| A1 | 20 (33%) | 37 (62%) | 1 (1.7%) | 2 (3.3%) | 0 (0%) | 60 |
| B1 | 3 (5.0%) | 51 (85%) | 0 (0%) | 6 (10%) | 0 (0%) | 60 |
| C1 | 22 (37%) | 27 (45%) | 1 (1.7%) | 9 (15%) | 1 (1.7%) | 60 |
| D1 | 22 (37%) | 27 (45%) | 3 (5.0%) | 8 (13%) | 0 (0%) | 60 |
| E1 | 16 (30%) | 26 (49%) | 0 (0%) | 6 (11%) | 5 (9.4%) | 53 |
| F1 | 24 (40%) | 30 (50%) | 4 (6.7%) | 2 (3.3%) | 0 (0%) | 60 |
| G1 | 15 (25%) | 36 (60%) | 2 (3.3%) | 7 (12%) | 0 (0%) | 60 |
| H1 | 19 (37%) | 26 (51%) | 1 (2.0%) | 5 (9.8%) | 0 (0%) | 51 |
| I1 | 20 (33%) | 27 (45%) | 3 (5.0%) | 10 (17%) | 0 (0%) | 60 |
| J1 | 22 (47%) | 16 (34%) | 3 (6.4%) | 6 (13%) | 0 (0%) | 47 |
| K1 | 3 (27%) | 3 (27%) | 3 (27%) | 2 (18%) | 0 (0%) | 11 |
| L1 | 12 (20%) | 38 (63%) | 0 (0%) | 10 (17%) | 0 (0%) | 60 |
| M1 | 19 (32%) | 32 (53%) | 6 (10%) | 3 (5.0%) | 0 (0%) | 60 |
| N1 | 15 (25%) | 37 (62%) | 4 (6.7%) | 4 (6.7%) | 0 (0%) | 60 |
| O 1 | 3 (7.7%) | 33 (85%) | 0 (0%) | 3 (7.7%) | 0 (0%) | 39 |
| P 1 | 8 (32%) | 12 (48%) | 5 (20%) | 0 (0%) | 0 (0%) | 25 |
| Q1 | 22 (37%) | 32 (53%) | 2 (3.3%) | 3 (5.0%) | 1 (1.7%) | 60 |
| R1 | 8 (16%) | 39 (78%) | 0 (0%) | 3 (6.0%) | 0 (0%) | 50 |
| S1 | 16 (27%) | 30 (50%) | 2 (3.3%) | 12 (20%) | 0 (0%) | 60 |
| T1 | 8 (19%) | 28 (65%) | 5 (12%) | 2 (4.7%) | 0 (0%) | 43 |
| U1 | 3 (6.5%) | 39 (85%) | 0 (0%) | 2 (4.3%) | 2 (4.3%) | 46 |
| V1 | 18 (45%) | 21 (53%) | 1 (2.5%) | 0 (0%) | 0 (0%) | 40 |
| W1 | 27 (45%) | 27 (45%) | 2 (3.3%) | 4 (6.7%) | 0 (0%) | 60 |
| X1 | 7 (12%) | 44 (73%) | 9 (15%) | 0 (0%) | 0 (0%) | 60 |
| Y1 | 19 (32%) | 30 (50%) | 6 (10%) | 5 (8.3%) | 0 (0%) | 60 |
| Z1 | 7 (18%) | 19 (50%) | 5 (13%) | 7 (18%) | 0 (0%) | 38 |
| A2 | 10 (17%) | 45 (75%) | 2 (3.3%) | 3 (5.0%) | 0 (0%) | 60 |
| B2 | 22 (43%) | 24 (47%) | 2 (3.9%) | 3 (5.9%) | 0 (0%) | 51 |
| C2 | 10 (25%) | 20 (50%) | 5 (13%) | 5 (13%) | 0 (0%) | 40 |
| **Total** | 420 (28%) | 856 (57%) | 77 (5.2%) | 132 (8.8%) | 9 (0.6%) | 1,494 |
